# Benchmarking Stochasticity behind Reproducibility: Denoising Strategies in Ta_2_O_5_ Memristors

**DOI:** 10.1021/acsami.5c00257

**Published:** 2025-04-19

**Authors:** Anna Nyáry, Zoltán Balogh, Botond Sánta, György Lázár, Nadia Jimenez Olalla, Juerg Leuthold, Miklós Csontos, András Halbritter

**Affiliations:** † Department of Physics, Institute of Physics, Budapest University of Technology and Economics, Muegyetem rkp. 3, H-1111 Budapest, Hungary; ‡ HUN-REN-BME Condensed Matter Research Group, Muegyetem rkp. 3, H-1111 Budapest, Hungary; § Stavropoulos Center for Complex Quantum Matter, Department of Physics and Astronomy, University of Notre Dame, Nieuwland Science Hall, Notre Dame, Indiana 46556, United States; ∥ Institute of Electromagnetic Fields, ETH Zurich, Gloriastrasse 35, 8092 Zurich, Switzerland

**Keywords:** memristor, resistive switching devices, 1/f-type noise, voltage-dependent noise, denoising strategies, noise tuning

## Abstract

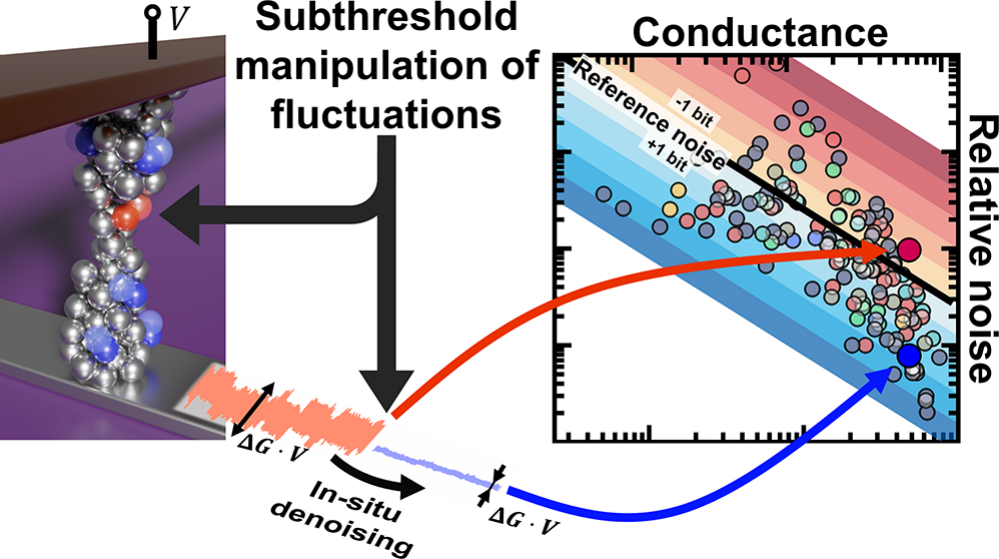

Reproducibility, endurance, driftless data retention, and fine resolution of the programmable conductance weights are key technological requirements against memristive artificial synapses in neural network applications. However, the inherent fluctuations in the active volume impose severe constraints on the weight resolution. In order to understand and push these limits, a comprehensive noise benchmarking and noise reduction protocol is introduced. Our approach goes beyond the measurement of steady-state readout noise levels and tracks the voltage-dependent noise characteristics all along the resistive switching *I*(*V*) curves. Furthermore, we investigate the tunability of the noise level by dedicated voltage cycling schemes in our filamentary Ta_2_O_5_ memristors. This analysis highlights a broad order-of-magnitude variability of the possible noise levels behind seemingly reproducible switching cycles. Our nonlinear noise spectroscopy measurements identify a subthreshold voltage region with voltage-boosted fluctuations. This voltage range enables the reconfiguration of the fluctuators without resistive switching, yielding a highly denoised state within a few subthreshold cycles.

## Introduction

Memristive devices are key candidates as artificial synapses for novel neuromorphic computing hardware applications.^1−8^ Among the most promising implementations are large-scale neural networks (NNs), where a memristive crossbar matrix encodes the synaptic weights of the NN in multilevel conductance states. This architecture performs the vector–matrix multiplication (VMM) in a *single* hardware-level operation step, as opposed to software NNs, where the VMM-based evaluation of a neural layer’s input requires ∼*N*^2^ operations, with *N* being the number of neurons in a layer.^9−11^ Recently, the energy-efficient solutions of several complex computational problems have been demonstrated using memristive crossbar arrays built of different material families.^12−14^ However, the next step from prototype applications to widespread commercialization depends heavily on the optimization of performance characteristics such as long-term data retention, cycle-to-cycle as well as device-to-device reproducibility, endurance, and the sufficiently high resolution of the conductance weights.^15^

Recently, Rao and co-workers demonstrated a high-performance memristive crossbar network reaching up to 11-bit resolution of the conductance weights.^15^ This was achieved by HfO_2_/Al_2_O_3_ and TaO_*x*_ filamentary memristive devices spanning an operation range of [*G*_min_, *G*_max_] = [50 μS, 4114 μS] in the achievable conductance levels and pushing the conductance resolution to (*G*_max_ – *G*_min_)/(2^11^ – 1) = 2 μS. This resolution was obviously limited by the internal fluctuations (noise) of the devices, which were successfully suppressed by a trial-and-error type noise reduction protocol.

**Figure 1:**
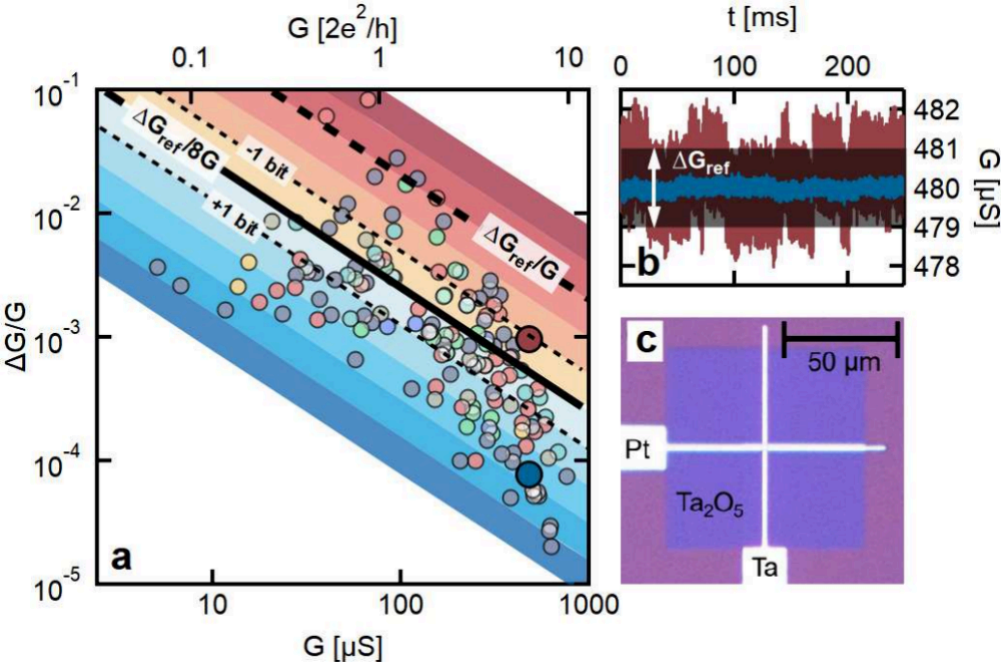
Steady-state noise characteristics of Ta/Ta_2_O_5_/Pt memristive devices. (a) Steady-state noise map presenting the Δ*G*/*G* relative conductance noise as a function of the *G* device conductance for 148 conductance states from 9 different devices (colored circles). These data are compared to the Δ*G*_ref_ = 2 μS reference conductance resolution from ref (15) (see the thick black dashed line). The peak-to-peak noise level is claimed to satisfy the reference conductance resolution below the Δ*G*_ref_/8*G* black solid line. Each cold (warm) color band represents an additional 1 bit of resolution improvement (deterioration) compared to the reference line. The top axis presents the conductance data in the units of the *G* = 2*e*^2^/*h* conductance quantum, highlighting the trend change in the noise map close to the conductance quantum unit. (b) Demonstration of the Δ*G*_ref_ = 2 μS reference conductance resolution and examples of noisy (dark red) and low-noise (blue) conductance signals. These dark-red and blue time traces correspond to 2 out of the 148 measurements on panel a sharing similar conductances but exhibiting fundamentally different relative noise levels (see the larger dark-red and blue dots in panel a). (c) Optical image of a Ta/Ta_2_O_5_/Pt cross-point device.

In some probabilistic computing schemes, noise can be exploited as a computational resource,^14,16−19^ but, as in the example above,^15^ in most applications the noise characteristics of memristive devices^20−33^ are indeed a crucial performance-limiting factor. For this reason, here, we perform a thorough noise analysis of similar Ta_2_O_5_ filamentary memristive devices (Figure 1c), addressing the following questions on the noise performance: How large is the general noise level in comparison to the above Δ*G*_res_ = 2 μS reference resolution, and how does the noise level depend on the conductance of the programmed state? Are the seemingly reproducible resistive switching characteristics reflected in a reproducible noise performance? Or more specifically: what is the device-to-device and cycle-to-cycle reproducibility of the noise level? Is the noise level stable below the threshold voltage of the resistive switching? To answer these questions, we have developed an elaborate full-cycle noise diagnostics protocol, where noise analysis is performed all along the resistive switching current–voltage [*I*(*V*)] characteristic, evaluating the noise data and the *I*(*V*) data from the same measurement. With this protocol, we reveal the cycle-to-cycle reconfiguration of the fluctuators, yielding a surprisingly large cycle-to-cycle variation of the noise level. Furthermore, we identify a non-steady-state subthreshold voltage region, where the fluctuators can be reconfigured by the applied voltage well below the resistive switching threshold. These discoveries provide deep insight into possible noise manipulation strategies, enabling an order of magnitude noise reduction by suitable subthreshold cycling.

## Results and Discussion

In the noise characteristics of a device, the so-called 1/*f*-type noise is often the dominant contributor in addition to the thermal noise floor.^34^ The former also appears in the steady state as a temporal fluctuation of the *G* device conductance with the Δ*G* standard deviation. At finite *V* readout voltage, this Δ*G* conductance fluctuation converts to a Δ*I* = Δ*G*|*V*| standard deviation of the measured current around its |*I*| average value. From this, the Δ*I*/|*I*| = Δ*G*/*G* relation follows, and these relative current or conductance fluctuations appear to be an adequate, voltage-independent measure of the noise characteristics.^20^ These considerations, however, are only valid for steady-state conductance noise measurements, assuming that (i) the Δ*G* conductance fluctuations are present at zero driving voltage and the application of the *V* readout voltage does not induce any further fluctuations, and (ii) the measurements are performed in the linear part of the *I*(*V*) curve, i.e., the *I* = *GV* Ohm’s law is satisfied with voltage-independent conductance. Relying on the above basics of steady-state noise measurements^20^ (see Section 1 in the Supporting Information for details), we first investigate the steady-state noise characteristics of our Ta/Ta_2_O_5_/Pt cross-point memristive devices (Figure 1c). See the Methods/Experimental Section section for the details of the sample fabrication and the noise measurement setup. With this approach, we map the device-to-device variations of the noise and investigate how the noise depends on the conductance of the device states selected in the context of multilevel programmability. Afterward, we introduce a protocol for full-cycle noise measurements, examining the variation of noise characteristics outside the steady-state regime. With the latter method, we investigate the cycle-to-cycle variation of the noise characteristics, and, more importantly, we explore the nonobvious voltage-induced variation and manipulation of the noise performance below the switching threshold.

### Steady-State Noise Map and Device-to-Device Noise Variation

In previous studies, it was demonstrated that noise maps, i.e., the steady-state Δ*G*/*G* relative device noise values plotted as a function of the *G* device conductance, are useful device fingerprints.^20−32^ The relevant transport mechanisms and key sources of the fluctuations are reflected by dedicated Δ*G*/*G* vs *G* dependencies, and a trend change in the Δ*G*/*G* vs *G* plot indicates a change in the transport mechanism. Figure 1a presents such a noise map based on the steady-state noise analysis of nine different Ta_2_O_5_ memristive devices. For each device, a broad range of different conductance states were programmed to map the conductance dependence of the noise characteristics as well as the state-to-state and device-to-device noise variation (see the circles in Figure 1a demonstrating measurements taken at 148 different conductance states, with the color shades representing the various devices). The noise characteristics are expected to exhibit a mostly conductance-independent Δ*G*/*G* vs *G* relation in the broken filamentary regimes, while a strongly conductance-dependent Δ*G*/*G* vs *G* relation is anticipated in the nonbroken filamentary regimes (see our review paper in ref (20)). In the case of atomic-sized filaments, the crossover is expected close to the *G*_0_ = 77.48 μS = 2*e*^2^/*h* quantum conductance.^20^ These core expectations are clearly confirmed by the observed tendencies in the Δ*G*/*G* vs *G* plot in Figure 1a (see the top axis in the units of 2*e*^2^/*h*): indeed, at *G* < 2*e*^2^/*h*, a mostly saturated relative noise amplitude is observed, while at *G* > 2*e*^2^/*h*, the relative noise amplitude exhibits a steep decrease with increasing conductance. In the latter case, the best fitting of the data is close to the Δ*G*/*G* ∼ *G*^–3^ trend, which can be explained by the effect of a single fluctuator in point-contact-like junction geometry, while the crossover at *G* ≈ 2*e*^2^/*h* in the noise map underpins the truly atomic-sized filamentary nature of the active region. See Figure S3 for a better visibility of the Δ*G*/*G* vs *G* trends as well as the fitting and discussion of the two corresponding transport regions.

The noise map of the Ta_2_O_5_ memristive devices (Figure 1a) also exhibits a remarkable, order-of-magnitude, device-to-device, and state-to-state noise variation for measurements sharing similar conductance values. To decide whether the observed noise levels are small or large, it is worth comparing the noise data with the outstanding Δ*G*_ref_ = 2 μS reference conductance resolution achieved in ref (15) over a similar conductance range. As the Δ*G* noise levels are normalized to the conductance, Δ*G*_ref_ should also be normalized to *G*, which is a line on the log–log plot (see the thick black dashed line in Figure 1a). Compared to this reference conductance resolution, the shaded areas illustrate other possible conductance resolutions with a factor of 2 (1 bit) resolution difference between the different shades. Note that, on the left axis, Δ*G* represents the standard deviation of the conductance, but to achieve the Δ*G*_ref_ conductance resolution, rather the peak-to-peak noise should be below the resolution limit. Therefore, as a safe margin, the relative noise should satisfy Δ*G*/*G* < Δ*G*_ref_/8*G*, i.e., to have a 3-bit better standard deviation of the noise as the envisioned reference resolution (see the black solid line). Exemplifying the same condition in the time domain, Figure 1b illustrates the temporal noise traces of the blue and red data points in Figure 1a, facilitating (blue) or conflicting (dark red) the reference conductance resolution.

The Δ*G*_ref_/8*G* line in Figure 1a clearly crosses the noise data, with a significant portion of the data points being below/above the reference line. This observation prompts the question whether a memristive device with Δ*G* > Δ*G*_ref_/8 is inherently too noisy for the reference resolution or whether a dedicated protocol could yield a significant noise reduction, keeping the conductance state practically unchanged. The study of non-steady-state noise will help to answer this question.

Prior to investigating noise reduction possibilities, we further analyze the shape of Δ*G*/*G* vs *G* tendencies in Figure 1a. Clearly, the transition between the nonbroken and broken filamentary regimes is the most critical, where the largest portion of the data points violates the condition for the reference resolution. This is understandable because the atomic-scale active region is very sensitive to any nearby fluctuations. At much lower conductances (*G* ≪ 2*e*^2^/*h*), the weak (mostly constant) Δ*G*/*G* vs *G* dependence of the broken filamentary regime helps to satisfy the reference resolution condition. Similarly, at *G* ≫ 2*e*^2^/*h*, the steep decay of Δ*G*/*G* with the widening of the filament also helps to keep the noise below the reference. All of these observations underpin the importance of plotting noise maps like Figure 1a because the satisfiability of the reference resolution condition strongly depends on the chosen conductance range.

### Full-Cycle Nonlinear Noise Spectroscopy

**Figure 2:**
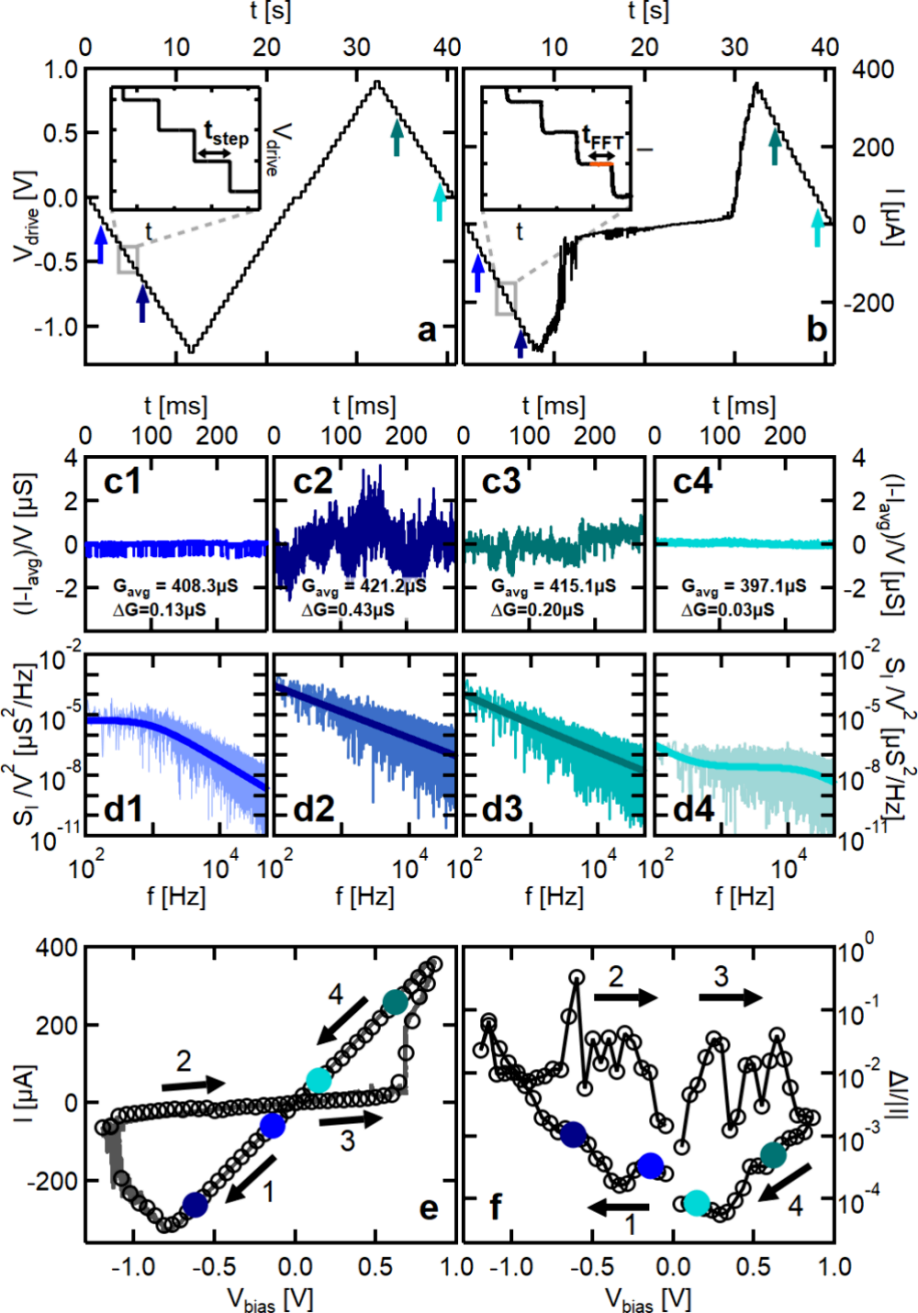
Full-cycle noise measurements. (a) Discretized voltage driving signal and (b) the corresponding measured current on a Ta/Ta_2_O_5_/Pt memristive device. The insets show the enlarged 2 s part of the voltage (current) traces with a full scale of 200 mV (80 μA). Due to the transients, the *I*_avg_ average current values and the *S*_*I*_(*f*) spectral densities are calculated for a *t*_FFT_ = 262 ms long (orange) part of the total *t*_step_ = 476 ms long current plateaus. (c1–c4) Demonstrative normalized current traces and (d1–d4) normalized noise spectra measured in the HCS at ±150 and ±650 mV (see the correspondingly colored arrows in panel a). Due to proper normalization (see the labels on the axes), these curves should look the same for steady-state fluctuations; i.e., the differences between the curves indicate voltage-manipulated noise variations. The corresponding average conductance and noise values are indicated on the panels. (e) *I*_avg_ vs *V*_bias_ curve and (f) Δ*I*/*I* vs *V*_bias_ full-cycle noise curves extracted from the discretized measurements (black circles). In panel e, the discretized *I*(*V*) measurement is compared with the previous continuous *I*(*V*) measurement (gray background curve). In panels e and f, the black arrows and numbers show the order of the quarter periods of the measurements, and the colored dots highlight the average current and relative noise values corresponding to the demonstrative curves in panels c1–d4.

So far, steady-state fluctuations have been investigated, which can be considered as a baseline for the read-out noise: regardless of the accuracy of the instrumentation used, the resolution of the conductance read-out cannot be better than steady-state noise. We then move beyond steady-state noise measurements, showing that noise benchmarking is possible all along the entire switching cycle. The scheme of our nonlinear noise spectroscopy measurements is demonstrated in Figure 2. As a key requirement, we wanted to match nonlinear noise and nonlinear *I*(*V*) data. However, the so-called time–voltage dilemma is well-known for memristive systems;^1,35−38^ i.e., the shape of the *I*(*V*) curve and especially the switching threshold strongly depend on the speed of the measurement. Accordingly, comparative *I*(*V*) and noise measurements should rely on voltage sweeps sharing the same amplitude and period time, or even better, the *I*(*V*) and noise data should be extracted from the same measurement.

In line with the above requirements, we first perform a traditional *I*(*V*) measurement with continuous triangular voltage driving for the initial characterization. Afterward, a discretized (stepwise) voltage sweep is performed (Figure 2a) using the same overall amplitude and period, while the current is measured (Figure 2b). The nontransient current response to the constant-level voltage plateaus (see the orange region in the inset of Figure 2b) provides *I*(*t*) temporal current traces at the various discrete voltage levels, from which the *I*_avg_ mean current is calculated by averaging, whereas the *S*_I_(*f*) spectral density of the current noise is obtained via Fourier transformation (see the Methods/Experimental Section section). Finally, the Δ*I*/|*I*| relative current fluctuation is obtained from the *S*_I_(*f*) noise spectrum by numerical integration:
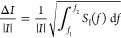
1where we consequently apply the *f*_1_ = 100 Hz and *f*_2_ = 50 kHz frequency limits. Example *I*(*t*) current traces and the related *S*_I_(*f*) noise spectra are respectively demonstrated in panels c1–c4 and d1–d4 of Figure 2. These measurements are related to voltage driving plateaus indicated by the corresponding colored arrows in Figure 2a,b.

From the current response to the stepwise voltage drive, one can plot a traditional *I*(*V*) curve at the discrete voltage levels (black circles in Figure 2e), which clearly shows bipolar switching between a high-conductance state (HCS) and a low-conductance state (LCS). This discretized *I*(*V*) curve perfectly matches the previously acquired conventional *I*(*V*) curve (gray line in the background), which was measured by a continuous triangular voltage sweep with the same overall amplitudes and period as the discretized *I*(*V*) curve. Note that both *I*(*V*) curves are plotted as a function of the *V*_bias_ = *V*_drive_ – *IR*_series_ voltage drop on the memristor; i.e., the voltage drop on the applied *R*_series_ = 110 Ω series resistor is subtracted from the drive voltage. From exactly the same discretized measurement, one can also plot the Δ*I*/*I* vs *V*_bias_ full-cycle noise curve (Figure 2f).

The representative full-cycle noise measurement in Figure 2 exhibits numerous remarkable features. First, the HCS displays a high degree of linearity up to the switching threshold voltage; i.e., the linearity condition of steady-state noise measurements is clearly satisfied. In the steady state, however, the Δ*G* standard deviation of the *G* = *I*/*V* conductance should be voltage-independent. This is strongly violated by Figure 2c1–c4, where the high-bias measurements (Figure 2c2,c3) exhibit much larger noise than the neighboring low-bias measurements (Figure 2c1,c4) (see the same measurements with a vertical scale highlighting the relative fluctuations in Figure S4). This means that in Figure 2c2,c3 the applied voltage excites a high level of fluctuations compared to the low-bias measurements even though the voltage remains below the switching threshold and *I*(*V*) is highly linear. The same feature is also demonstrated by the corresponding colored points in Figure 2f. Furthermore, in the low-bias measurements (Figure 2c1,c4), the noise spectrum (Figure 2d1,d4) is dominantly Lorentzian-type, which is characteristic of a single dominant fluctuator with a specific fluctuation time constant.^20^ As a sharp contrast, the high-bias noise measurements (Figures 2d2,d3) display 1/*f*-type spectra, which is characteristic of a large number of relevant fluctuators with a broad distribution of fluctuation times.^20^ This indicates the voltage-induced activation of a large number of fluctuators. See Section 2 in the Supporting Information for more details on the decomposition of the spectra to Lorentzian and 1/*f*-type contributions.

**Figure 3:**
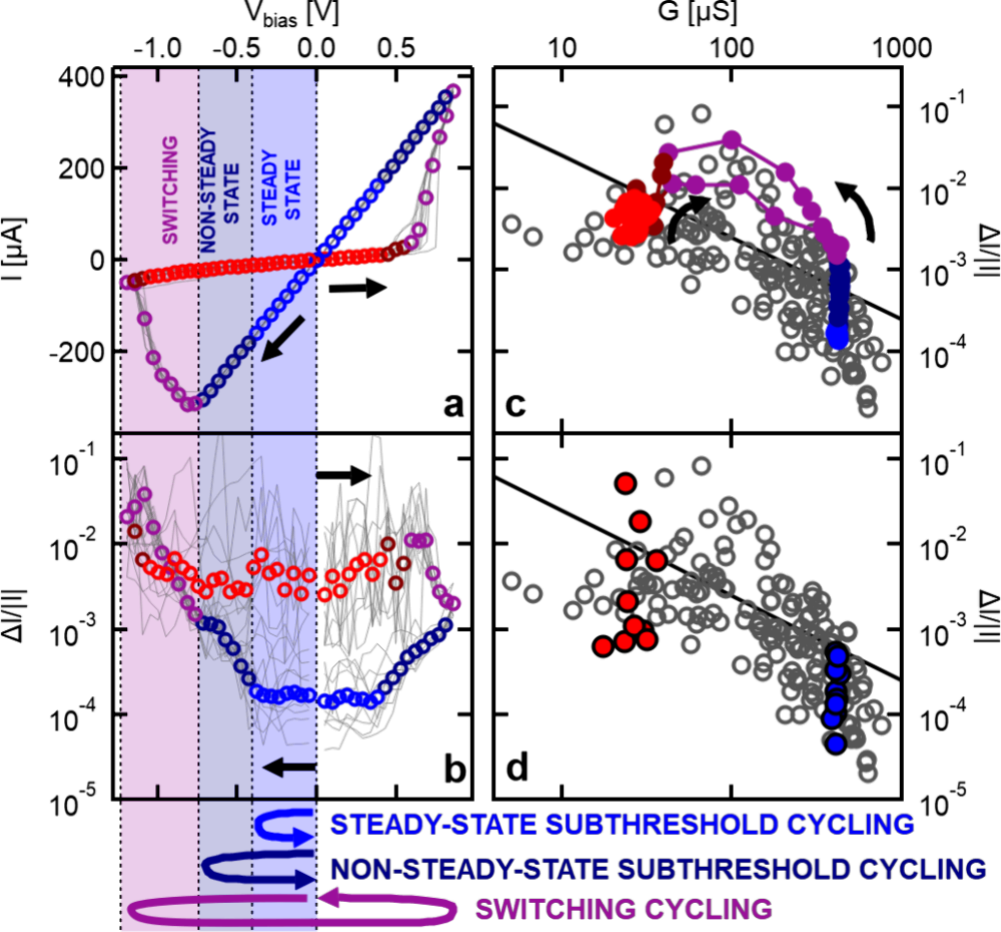
Average full-cycle noise characteristics and cycle-to-cycle noise variations for repeated switching cycles. (a and b) Results of 10 subsequent discretized full-cycle noise measurements (gray lines in panel b), the average full-cycle noise curve for these measurements (color circles in panel b), and the related discretized *I*(*V*) curves (gray lines in panel a) and average *I*(*V*) curves (color circles in panel a). The individual gray curves exhibit highly reproducible conductance states in the *I*(*V*) characteristics but huge variations in the relative noise. The entire voltage region of the measurement is split into three characteristic regions: the steady-state region (blue and red circles for the HCS and LCS), where the average relative noise is mostly constant, the non-steady-state subthreshold region (dark-blue and dark-red circles for the HCS and LCS) with voltages below the switching threshold but increased relative noise compared to the steady state, and the switching region (purple circles), where the resistive switching happens. For the HCS → LCS switching, these regions are also illustrated by the correspondingly shaded background areas. The colored arrows below illustrate the three cycling strategies to manipulate the steady-state noise. (c) Average relative noise curve from panel b replotted in comparison to the steady-state noise map of Figure 1a, such that the horizontal axis is calculated as *G* = *I*/*V*_bias_ for the average relative noise curve. (d) Cycle-to-cycle variation of the HCS (blue) and LCS (red) steady-state relative noise levels measured between the 10 subsequent switching cycles at low (150 mV) voltage levels. This cycle-to-cycle noise variation is compared to the device-to-device and state-to-state noise variations reproduced from Figure 1a (gray circles). The solid gray lines in panels c and d indicate the Δ*G*_ref_/8*G* reference resolution.

Similar features are also found along repeated switching cycles, as demonstrated in Figure 3a,b. Here the gray curves in the background display 10 subsequent *I*(*V*) curves (a) and full-cycle noise curves (b). The *I*(*V*) curves exhibit a remarkable cycle-to-cycle reproducibility, which contrasts the noise measurements, where a huge cycle-to-cycle variation is experienced (see Section 4 in the Supporting Information for a more detailed analysis and figure). The colored circles represent the average *I*(*V*) curve and average full-cycle noise curve for the 10 cycles. These average curves unambiguously show multiple characteristic regimes: (i) a steady-state regime at low voltages with voltage-independent relative noise levels, (ii) a non-steady-state regime at slightly increased voltages still in the linear, with a nonswitching conduction regime of the current–voltage characteristics exhibiting an order of magnitude increase of relative noise, and (iii) a switching regime where the resistive transition occurs. The color coding of the corresponding data points for set/reset transitions is (i) red/blue, (ii) dark red/dark blue, and (iii) purple. The relative noise increase in the non-steady-state regimes (dark red and dark blue) is attributed to voltage-induced activation of ionic motions around the active region, but this is not yet a resistive switching, only a fluctuation with mostly unchanged mean conductance. In this sense, the non-steady-state region is considered to be a precursor regime: the increasing noise forecasts the proximity of the switching.

To put the full-cycle noise data into perspective, Figure 3c plots the average relative noise data (red, dark-red, blue, dark-blue, and purple curve segments in Figure 3b) on top of the steady-state noise map reproduced from Figure 1a (gray circles). Compared to the strong, order-of-magnitude device-to-device variation of the gray steady-state noise data, the steady-state regime of the average voltage-dependent noise curve (red and blue circles) exhibits a smaller variation in both the relative noise and conductance. The conductance of the LCS corresponds to a more oxygen-saturated conducting filament with a transport deviating from metallic conduction and more prone to instabilities, which explains the broader variation in conductance. By definition, the non-steady-state regime is the voltage range, where the conductance is mostly unchanged, but the noise significantly deviates from the steady-state noise. Accordingly, the corresponding dark-red and dark-blue non-steady-state noise data are positioned above the steady-state noise data in Figure 3c. Finally, the noise data in the switching region (purple) do not grow above the device-to-device variation of the steady-state noise data, but the purple points are positioned around the largest possible steady-state noise values at the given *G* = *I*/*V*_bias_ conductance of the actual point on the switching curve.

### Cycle-to-Cycle Noise Variation

In addition to the voltage-induced excitation of non-steady-state fluctuations, the example measurements in Figure 2 display an additional remarkable feature. At the beginning of the full cycle (Figure 2c1), the *G*_avg_ = 408.3 μS conductance is accompanied by Δ*G* = 0.13 μS conductance noise. At the end of the full cycle (Figure 2c4), the conductance returns to a very similar value (≈3% conductance change); meanwhile, the noise reduces by more than a factor of 4 (see Δ*G* = 0.03 μS in Figure 2c4), and a comparison of the light-blue and light-cyan points is given in Figure 2f. This means that a single switching cycle remarkably manipulates the dominant fluctuators and related noise performance.

The related cycle-to-cycle noise variation is even better displayed in Figure 3d, where the blue and red points demonstrate the steady-state (low-bias) noise values measured between the subsequent switching cycles of Figure 3a,b compared with the device-to-device and state-to-state variation of the steady-state noise values (gray circles reproduced from Figure 1a). Even though the repeated switching cycles with seemingly reproducible *I*(*V*) curves are intended to restore the same device states in each cycle, it is clear that the cycle-to-cycle variation of the steady-state noise values spans the same wide range as the device-to-device and state-to-state variation.

The previous observations mean that the seemingly reproducible switching process yields the reconfiguration of the fluctuators along the switching; i.e., a completed switching cycle yields a conductance very similar to that of the previous cycle, while the fluctuations of the active region completely change. This can cause even an order-of-magnitude decrease of the steady-state noise from one cycle to the other but a similarly large increase as well, which can be interpreted by switching OFF or ON a highly dominant fluctuator along a resistive switching cycle. This cycle-to-cycle noise variation does not allow a deterministic denoising strategy, but by trial-and-error, along ∼10 switching cycles, one can find a device state for which the steady-state noise is close to the bottom end of noise levels’ device-to-device variation; i.e., the noise of the actual state is close to the smallest possible noise value for the given device pool. Accordingly, the such-adjusted low-noise state is applicable as a high-resolution synaptic weight in a NN.

### Subthreshold Denoising

In the following, we analyze the possibility of subthreshold denoising on the Ta_2_O_5_ cross-point devices, following the voltage-cycling strategies illustrated at the bottom of Figure 3. The bottom strategy (switching cycling) illustrates just the above-described scheme, where the steady-state noise of a device can be manipulated throughout repeated switching cycles. Alternatively, one can ramp up and down the applied voltage, always staying below the switching threshold, and study how the steady-state noise is manipulated by such *subthreshold cycling measurements*. By a definition of the steady state, no major variation of the noise properties is expected if a *steady-state subthreshold cycling* is performed, i.e., if the applied voltage cycle does not exceed the steady-state region. A *non-steady-state subthreshold cycling* may already yield considerable noise variation, offering the possibility for subthreshold denoising. These schemes are discussed in the following by demonstrating a noise benchmarking protocol that provides comprehensive insight into the noise properties along the above cycling strategies.

**Figure 4:**
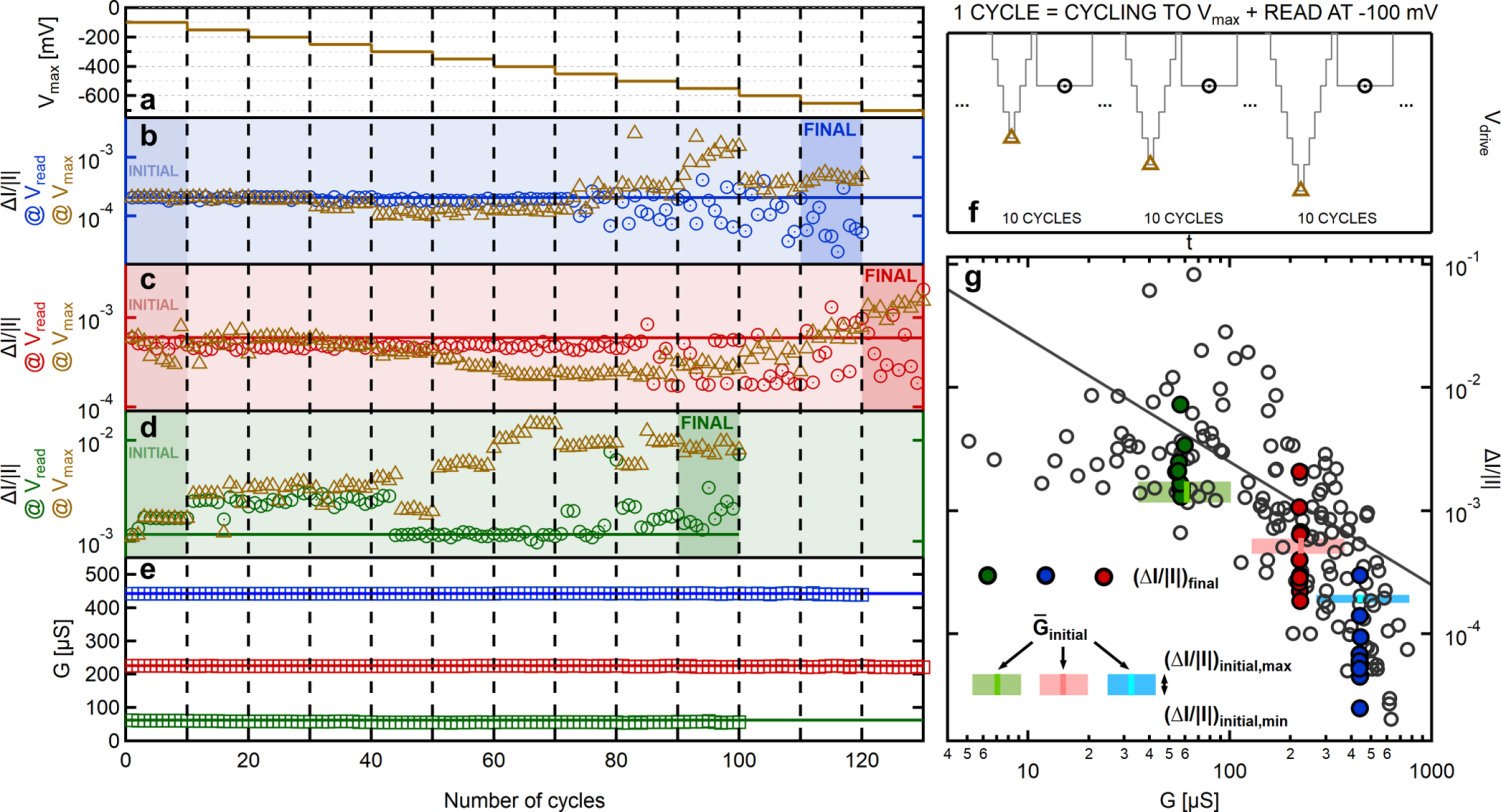
Subthreshold cycling measurements demonstrating noise manipulation/noise retention in the non-steady-state/steady-state regimes. One cycle of the subthreshold cycling consists of a discretized voltage sweep down to the maximum negative drive amplitude (*V*_max_) and back to zero voltage and a subsequent 5 s long low-voltage (*V*_read_ = −100 mV) read-out plateau (f). Altogether 130 cycles are performed, with 10 cycles sharing the same *V*_max_ and increasing *V*_max_ afterward as shown in panel a. (b–d) Relative noise measured along the various cycles at the *V*_max_ peak voltage (triangles) and the *V*_read_ readout voltage after the cycle (circles). The three panels respectively correspond to device states with 5.7*G*_0_, 2.9*G*_0_, and 0.8*G*_0_ (442, 225, and 62 μS) conductances; the conductance stability along the cycles is demonstrated in panel e. The horizontal solid colored reference lines in panels b–e show the relative noise (conductance) at the beginning of the subthreshold cycling process. (g) Variation of the relative noise levels along the subthreshold cycles in comparison to the device-to-device noise variation reproduced from Figure 1a. The blue, red, and green symbols respectively correspond to the measurements in panels b–d. The vertical extent of the blue, red, and green shaded areas demonstrates the noise variation for the smallest amplitude *V*_max_ = −100 mV subthreshold cycles. The increased width of the shading is purely for better visibility, with the vertical lines in the middle showing the average conductance for the 10 low-amplitude cycles. The blue, red, and green circles demonstrate the variation of the readout relative noise levels for the final 10 high-amplitude subthreshold cycles (see the *final* labels on panels b–d). The thick solid gray line indicates the Δ*G*_ref_/8*G* reference resolution.

We analyzed the variation of the steady-state noise with the subthreshold measurement protocol. First, a long read-out noise measurement is executed at *V*_read_ = −100 mV. Next, the voltage is ramped up toward the negative polarity in a stepwise fashion to a *V*_max_ level that is still below the switching threshold. Afterward, the voltage is ramped down to zero in a stepwise fashion. Finally, another noise measurement is performed at *V*_read_, as illustrated through the corresponding driving signal in Figure 4f. The results of such measurements are summarized in Figure 4a–e. The horizontal axis shows the number of executed cycling periods, while Figure 4a presents the *V*_max_ voltage reached along the given cycle. First, a *V*_max_ value of −100 mV is applied. Each cycling amplitude is repeated 10 times to observe the cycle-to-cycle variation at a given voltage amplitude, and then *V*_max_ is increased by −50 mV toward the negative polarity. Negative voltages were chosen based on the findings evident in Figure 3b, which shows that the subthreshold non-steady-state noise increase is most pronounced under negative polarity. This schematic is repeated for 130 cycles in total, reaching *V*_max_ = −700 mV. The latter value relies on our experience with a large number of devices. It represents a voltage level where the switching threshold is not yet reached; i.e., the steady-state conductance is mostly unchanged, but the steady-state noise regime is well exceeded. Eventually, only the part exhibiting stable conductance is included in the analysis. Practically, this means that cyclings leading to a >10% change in the conductance are excluded. This was the case for the measurements of Figure 4b,d, where the cyclings with maximal amplitudes above *V*_max_ = −650 mV and −550 mV resulted in an enhanced change of the conductance.

Such cycling periods are presented on three different conductance states (Figure 4b–d), well representing the entire conductance region of the usual measurements. The experiments yield two useful quantities for further evaluation: (i) the steady-state relative noise level after a certain cycling period is evaluated along the *V*_read_ read-out plateau (read-out noise demonstrated by colored circles with dots in the middle in Figure 4b–d); (ii) the relative noise is also evaluated at each voltage plateau of the cycling, and the highest voltage (*V*_max_) is used in this analysis, as demonstrated by the gold triangles in Figure 4b–d. As a reference, the conductance is also evaluated after each cycle at *V*_read_, as demonstrated by the colored squares in Figure 4e using the same colors for the various conductance states as those in the noise data in Figure 4b–d. The initial steady-state relative noise and conductance values are indicated by solid horizontal colored lines to emphasize any relative change.

We emphasize that the conductance (Figure 4e) is extremely stable along all of these subthreshold voltage cycles. The noise variation measured at *V*_max_ (triangles in Figure 4f) is not necessarily monotonic, but a general increasing trend is identified in accordance with the non-steady-state noise region in Figure 3. This is attributed to the tendency that a non-steady-state voltage is likely to excite further fluctuators compared to the steady state, but sometimes it is also possible that the applied voltage pushes a certain fluctuator to a state where it stops fluctuating or, alternatively, it modifies its fluctuation frequency, which also alters the integrated current fluctuation. Section 5 in the Supporting Information presents a detailed example of such voltage-induced tuning of a dominant fluctuator that can lead to an initial decrease of noise before the increasing tendency of the non-steady state is observed.

The read-out noise (colored circles in Figure 4g) is mostly constant until *V*_max_ = −450 mV or *V*_max_ = −500 mV voltage amplitudes, corresponding to device states with 5.7*G*_0_ and 2.9*G*_0_ conductances, which is apparent in panels b and c of Figure 4, respectively. At even higher *V*_max_, the read-out noise values do not show a general increasing tendency, but rather a stochastic variation between the subsequent subthreshold cycles is observed, such that the actual noise values can even be significantly smaller than the initial read-out noise. The results in Figure 4d are obtained at a lower conductance of 0.8*G*_0_ (green), where the device is less stable than at the above-mentioned higher conductances. Around the quantum conductance unit, the smallest rearrangements in the conductive filament strongly influence the overall conductance and the steady-state read-out noise. Such instabilities hinder the unambiguous identification of the steady-state and non-steady-state regimes. Nevertheless, the results do suggest an onset of the non-steady state around −400/–450 mV with a considerably increased maximal amplitude noise relative to the initial values and, more apparently, an increased cycle-to-cycle variation of the read-out noise.

The results of Figure 4b–d can also be summarized in comparison to the reference figure on the device-to-device steady-state noise variation (gray circles in Figure 4g). In the same figure, the vertical extent of the light-blue, light-red, and light-green shaded areas encloses the intervals, where the relative read-out noise values scatter for the initial 10 cycles in Figure 4b–d, i.e., for the measurements with *V*_max_ = −100 mV. These noise values span a significantly narrower noise interval than the device-to-device variation represented by the gray circles. This means that the low voltage cycling keeps the device’s noise mostly stable. As a sharp contrast, the blue, red, and green circles in Figure 4g exhibit the read-out noise values during the final 10 cycles in Figure 4b–d, where the *V*_max_ voltage amplitudes reach the maximal values in the non-steady-state regime. These noise values span a noise interval similar to that of the device-to-device variation at the same conductance. All of these observations demonstrate that an initial high noise of a memristive device (like the red point and red curve in Figure 1a,b) is not an immutable property, and not even complete switching cycles are needed to tune the noise. With subthreshold cycling, the full available noise range can be traversed with a few voltage sweeps and a lower noise state can be set.

## Conclusions

We have investigated the noise properties of Ta_2_O_5_-based cross-point memristive devices by comparing their noise levels with that of the Δ*G*_ref_ = 2 μS reference conductance resolution obtained in ref (15). We first investigated the steady-state readout noise levels using low-bias measurements. This analysis revealed a clear trend change in the relative noise vs conductance map, with a strongly conductance-dependent/mostly conductance-independent Δ*G*/*G* vs *G* relation in the unbroken/broken filamentary regimes. At the high- and low-conductance ends of this noise map, the observed noise levels mostly satisfy the targeted reference conductance resolution, while in the intermediate conductance range, noise levels are often observed above the reference due to the extreme sensitivity of atomic-scale filaments to nearby atomic fluctuations. In addition to these noise trends, huge order-of-magnitude device-to-device and state-to-state noise variation was also observed.

Moving beyond the steady state and to explore the voltage-induced manipulation of the fluctuators, we introduced a protocol to characterize the noise along the full switching cycle. This analysis highlighted a remarkable voltage-induced noise increase below the switching threshold voltage, even when a highly linear subthreshold *I*(*V*) curve is observed. This has been attributed to a precursor effect, i.e., the gradual mobilization of additional fluctuators as the switching process approaches. The full-cycle noise measurements also reveal a surprisingly large variation in readout noise levels from cycle to cycle, explained by the activation or deactivation of the fluctuators during the switching process. This very strong cycle-to-cycle variation in noise levels reaches the level of device-to-device noise variation, which contrasts with the negligible cycle-to-cycle variation of the *I*(*V*) curve.

Finally, we exploited the precursor noise phenomenon to manipulate the device noise without switching. For this purpose, we have presented a non-steady-state subthreshold voltage cycling method for manipulating the noise level over the range of the device-to-device noise variation and also identified a steady-state voltage regime suitable for stable operation while preserving the initial noise levels. All of these results show that a relatively high initial noise level of the investigated devices is not an immutable property; rather, with appropriately designed voltage cycles, significant noise reduction can be achieved without the need to switch the device.

In this work, noise reduction was investigated specifically for Ta_2_O_5_ filamentary memristors. However, the presented full-cycle measurement technique allows an in-depth noise analysis of a wide range of memristors, offering fundamental information on the overall noise levels and their conducance dependence, as well as on the cycle-to-cycle reproducibility and voltage tunability of noise in different memristive material species. We also note that the presented subthreshold denoising cycles are based on a trial-and-error protocol. This imposes an extra overhead on the circuitry because each weight update must be accompanied by fine denoising cycles, which also require a sensitive noise readout after each cycle. Therefore, this useful noise reduction option still leaves room for other approaches, such as noise engineering through material selection and optimization,^17,22^ temperature reduction or temperature annealing,^26,28,33,39^ or noise reduction through postelectroforming training cycles.^32^ We are confident that the combination of all of these approaches will yield detailed know-how on the efficient noise engineering of memristive devices.

## Methods/Experimental Section

### Device Fabrication

The detailed noise spectroscopy measurements and evaluation are performed on Ta/Ta_2_O_5_/Pt cross-point devices.^40^ The sample is fabricated on top of a SiO_2_ substrate where a Ti adhesive layer of 10 nm and a Pt bottom layer of 40 nm thickness are deposited by electron-beam evaporation. Subsequently, the 5-nm-thick Ta_2_O_5_ layer is sputtered by reactive high-power-impulse magnetron sputtering. Finally, the 65-nm-thick Ta top layer and an additional Pt capping layer are sputtered on top of the Ta_2_O_5_ layer.

### Noise Measurement Setup

**Figure 5:**
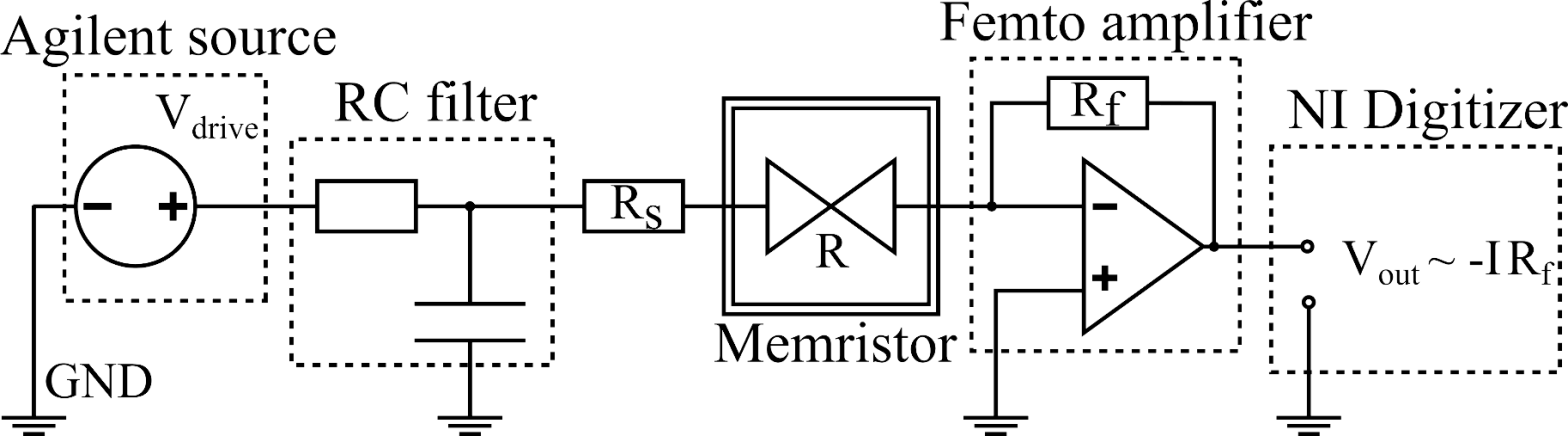
Noise measurement setup. The arbitrary waveform generator outputs the voltage through the RC low-pass filter, the sample, and the instrumental series resistances. The current on the sample is amplified by the current amplifier with a gain-dependent feedback resistor and finally measured with a high-resolution digitizer.

The Ta/Ta_2_O_5_/Pt cross-point devices are investigated in a Faraday cage and with shielded cables in order to eliminate external instrumental noise appearing in the measurements. The circuit diagram of the noise measurement setup is depicted in Figure 5. An Agilent 33220A arbitrary waveform generator serves as a voltage source with a simple low-pass RC filter on the output. The latter decreases the output noise of the generator and can be tuned in accordance with the time scale of the actual measurement. The device current is amplified by an Femto DLPCA-200 current amplifier and recorded by a National Instruments PXI-5922 digitizer that offers a 6 MHz aliasing-free bandwidth. The *R*_series_ series resistor has a current limiting role in the low-resistance states of the devices.

From the measured *I*(*t*) time traces, the spectral density of the current noise is evaluated in software according to the
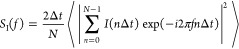
2relation, where Δ*t* is the time between subsequent sampling events, and the averaging is performed for different time traces, each containing *N* data points. All measurements include a zero-bias noise spectrum, which is subtracted from all biased spectra; thus, only the excess noise is evaluated.

With this method, the noise spectra are evaluated within the 100 Hz to 50 kHz frequency range, which we use as a standard for our measurements in order to ensure comparability of noise characteristics recorded for different parameters, e.g., different memristor resistances. The upper limit is fundamentally set by the limited bandwidth of the current amplifier in the high-gain (10^7^ V/A) mode, which is used at high resistances. On the other hand, the 100 Hz bottom limit allows a full-cycle noise measurement with ≈80 subsequent voltage plateaus per cycle such that the overall measurement time of the full cycle remains reasonable (≈40 s).

## Supporting Information Available

Representative example of steady-state noise characterization of a memristive device, additional information about the decomposition of the noise spectra into 1/*f*^γ^ and Lorentzian contributions, further evaluation and discussion of the steady-state noise map, in-depth analysis of the conductance and relative noise evolution preceding and during the reset transition, and an example of a non-steady-state cycling tuning a dominant fluctuator (PDF)
