# Supplementary material for: Benchmarking Stochasticity behind Reproducibility: Denoising Strategies in Ta2O5 Memristors
Source: ACS Appl Mater Interfaces. 2025 Apr 19;17(17):25654–62. doi: 10.1021/acsami.5c00257 (PMC12051165; doi:10.1021/acsami.5c00257)
Supplement: Supplementary file 1 — am5c00257_si_001.pdf [file am5c00257_si_001.pdf]

Supporting information
Benchmarking Stochasticity Behind Reproducibility: Denoising Strategies in Ta_2O_5 Memristors

Anna Nyáry[a,b,c], Zoltán Balogh[a,b], Botond Sánta[a,b], György Lázár[a], Nadia Jimenez Olalla[d], Juerg Leuthold[d], Miklós Csontos[d], and András Halbritter*[a,b]

[a] *Department of Physics, Institute of Physics, Budapest University of Technology and Economics, Műegyetem rkp. 3., H-1111 Budapest, Hungary*

[b] *HUN-REN-BME Condensed Matter Research Group, Műegyetem rkp. 3., H-1111 Budapest, Hungary*

[c] *Stavropoulos Center for Complex Quantum Matter, Department of Physics & Astronomy, Nieuwland Science Hall, Notre Dame, IN 46556 USA*

[d] *Institute of Electromagnetic Fields, ETH Zurich, Gloriastrasse 35, 8092 Zurich, Switzerland*

**Corresponding author: halbritter.andras@ttk.bme.hu*

1 Steady-state noise measurements

A representative example of the steady-state $1/f$-type noise spectroscopy measurement on a $Ta/Ta_2O_5/Pt$ memristive switching device is demonstrated in Fig. S1a-d. Panel (a) depicts a representative $I(V)$ curve from a stable, reproducible switching. The noise measurements are performed in the low-conducting state (LCS) and the high-conducting state (HCS), denoted in all panels by red and blue colors, respectively, at applied voltages featuring linear characteristics satisfying Ohm's law. Panels (b) and (c) show the raw noise spectra obtained at the LCS and HCS at V_{drive} voltages indicated in the legends. The S_I spectra in both states exhibit a significant contribution from a single dominant fluctuator near the smallest constriction to the $1/f$-type noise from an ensemble of remote fluctuators. See more on the decomposition of the noise spectra in Section 2. The black spectra are the base noise spectra at zero bias composed of the instrumental noise at low frequencies and the Johnson-Nyquist noise background dominating at high frequencies. The blue and red dashed horizontal lines signify the expected base noise levels calculated from the current and voltage noise of the amplifier and the Johnson-Nyquist noise given by the device resistance and temperature. In order to obtain the $(\Delta I)^2$ mean squared deviation of the current, the corresponding zero-bias base spectrum is subtracted from each voltage-driven noise spectrum, then $(\Delta I)^2$ is given by the integral

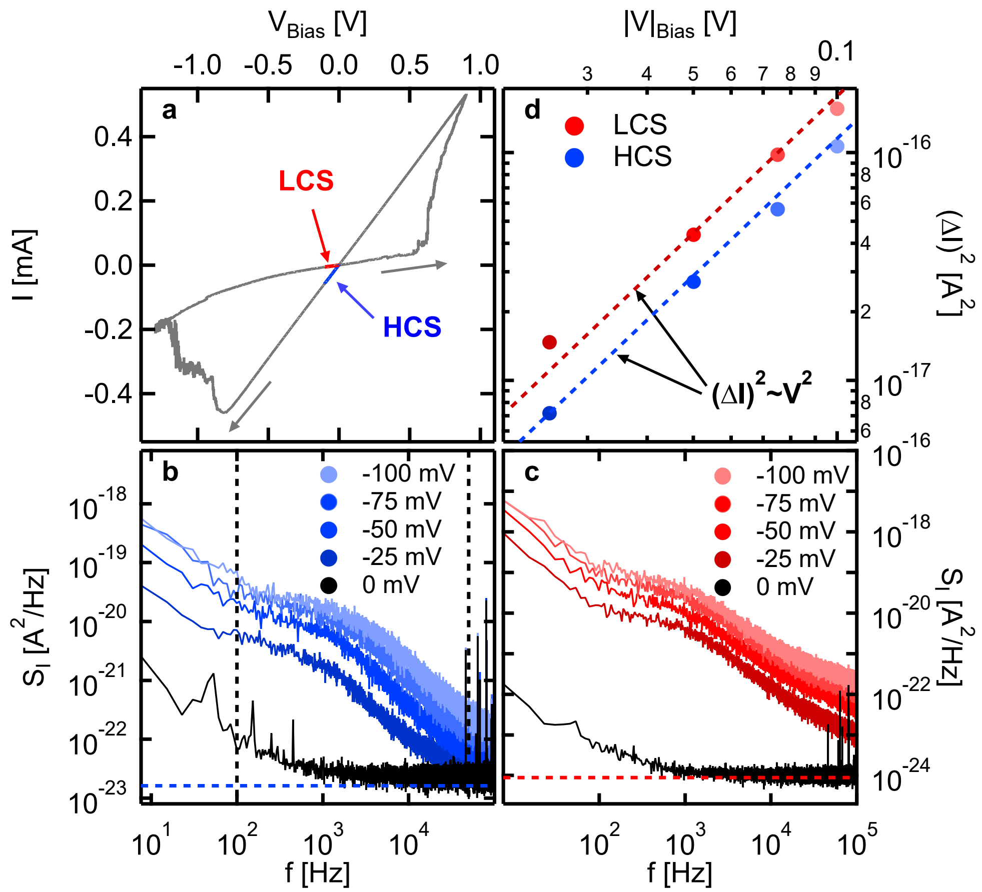

Figure S1 Steady-state noise characterization of a Ta/Ta$_2$O$_5$/Pt crosspoint switching device. (a) Representative switching in a device with colored sections of the 7.6 G_0 HCS (blue) and 0.65 G_0 LCS (red) indicating the low-voltage region used in the steady-state noise measurements. (b-c) Voltage dependence of the noise spectral density obtained at the HCS and LCS with the corresponding base noise spectra (black). The horizontal dashed lines represent the expected base noise level calculated from instrumental and Johnson-Nyquist noise. (d) The steady-state mean squared deviation of the current calculated from the spectral integral between $f_1 = 100$ Hz and $f_2 = 50$ kHz frequency limits (see vertical dashed lines in panel (b)) which follows the expected V^2-dependence (see guide to the eye with colored dashed lines).

between the predefined $f_1 = 100$ Hz and $f_2 = 50$ kHz frequencies (see dashed vertical lines in panel (b)). In the linear I(V) characteristic regime, based on Ohm's law, quantitatively $(\Delta I)^2 \sim V^2$ is expected for steady-state resistance (conductance) noise, as it is observed in panel (d) for both HCS and LCS.

2 Decomposition of noise spectra

At any conductance state, the obtained noise spectra can exhibit contribution from a single dominant fluctuator near the smallest constriction to the $1/f$-type noise from an ensemble of remote fluctuators, which manifests as a Lorentzian-type spectrum superimposed on $1/f$-type spectrum. In

most cases, it seems reasonable to assume that the noise adds up from a pure $1/f^{\gamma}$ and a Lorentzian spectrum:

$$S_I(f) = S_{I,1/f}(f) + S_{I,\text{Lorentzian}}(f) = \beta \cdot \frac{1}{f^{\gamma}} + \frac{A \cdot \tau}{1 + (2\pi f)^2 \tau^2}. \quad \text{(S1)}$$

The spectra can be fitted corresponding to this description on the log-log scale, and the resulting analytical sum function can be used to calculate the noise-to-signal ratio. By analyzing the fitting, the analytical integral of the $1/f$-type and Lorentzian contributions can be calculated separately to obtain even more information about the behavior of the fluctuators. Some examples of typical mixed noise spectra in Ta_2O_5-based memristor are demonstrated in Fig. S2. The legends of the

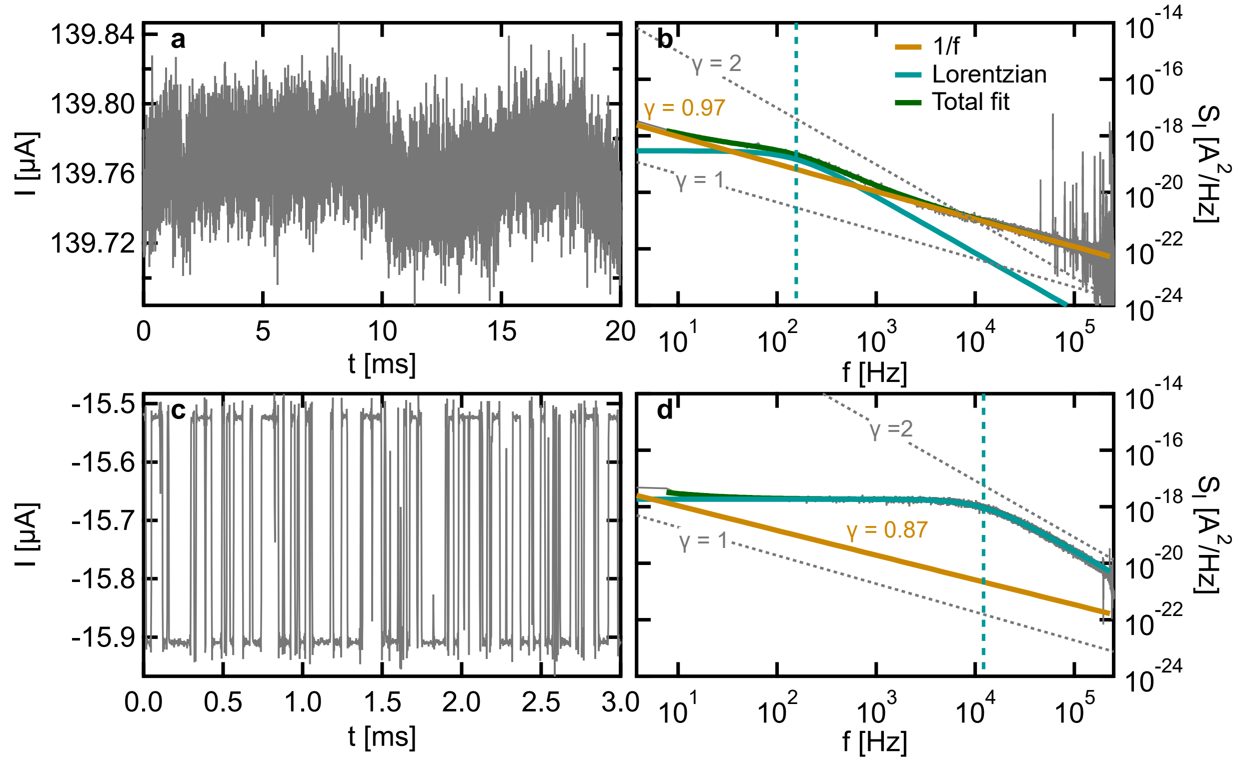

Figure S2 Gallery of mixed spectra and segments of the corresponding current signals measured in Ta/Ta_2O_5/Pt memristor. The spectra are decomposed to a Lorentzian spectrum of a significantly contributing fluctuator (cyan) superimposed on the $1/f$-type noise of a remote ensemble of fluctuators (gold). The final parameters of the fit are presented in the legends with the A Lorentzian magnitude. (a-b) The spectral density shows a Lorentzian emerging from the $1/f$-type spectrum with a characteristic low-frequency time scale. (c-d) The current signal shows clear indications of an RTN, and correspondingly, the Lorentzian spectrum dominates in the full frequency range, hindering the confident fitting of the $1/f$-type contribution.

spectra show the parameters of the decomposed $1/f$-type and Lorentzian spectrum. Panels (a)-(b) introduce a mixed spectrum together with a segment of the corresponding current signal. When the fluctuator contributes more significantly to the transport, the current signal shows the typical

random telegraph noise (RTN) characteristics and a corresponding dominant Lorentzian spectrum, shown in panels (c)-(d).

3 Conductance dependencies of steady-state noise

The steady-state noise map of Ta/Ta_2O_5/Pt memristive devices is presented in Fig. 1 in the main text. Here, we illustrate the same data set in Fig. S3 with each of the 9 independent devices colored differently. The figure shows that the noise map serves as a unique fingerprint for the device, with consistent behavior across independent devices of the same design. In this section, we focus on the $\Delta G/G$ vs. G dependencies observed in the noise map that provide insights into the relevant transport mechanisms and the sources of the fluctuations.

The noise map shows a considerable variation of the relative noise with the device conductance: a rather conductance-independent relative noise ($\sim G^{0.02}$) below 0.5 G_0, and a steep decrease above 2 G_0 ($\sim G^{-2.24}$). The possible conductance dependencies of the relative steady-state noise levels are discussed in the review paper Ref. 1 where it is demonstrated that in a tunneling barrier-like junction, the conductance is an exponential function of the possible fluctuating parameters (like the width or the height of the barrier), and this exponential dependence yields a mostly conductance-independent relative noise, which is the case for the studied Ta_2O_5 crosspoint devices at $G < 1\,G_0$ conductances.

In the following, we consider the non-broken filamentary regime of a diffusive point-contact relevant in the high conductance regime of $G > 1\,G_0$ conductances. The point-contact geometries depicted in the insets of Fig. S3 provide illustrations for the geometry of the resistive switching cells with two extreme cases of fluctuator distribution: fluctuators distributed in the entire volume (lower inset) and single fluctuator in the narrowing (upper inset). The type of assumed geometry is a narrowing, which has a characteristic diameter d at the bottleneck, but it does not have a characteristic length, the junction diameter is the only determining dimension.

In the limiting case that is also discussed in Refs. 1–4, fluctuators distributed in the entire volume of the point-contact are considered and the conductance is approximated by the Maxwell formula[5,6], $G_{\mathrm{PC}} = \sigma \cdot d$, where σ is the conductivity. Based on the model considerations, the relative noise in the case of fluctuators distributed in the entire point-contact (see lower illustration and corresponding gray dashed guide to the eye line) follows $\Delta G_{\mathrm{PC}}/G_{\mathrm{PC}} \sim G_{\mathrm{PC}}^{-3/2}$ conductance dependence. Ref. 3 showed this exact conductance dependency of the relative steady-state noise for Ta/Ta_2O_5/PtIr scanning tunneling microscope (STM) point-contact devices.

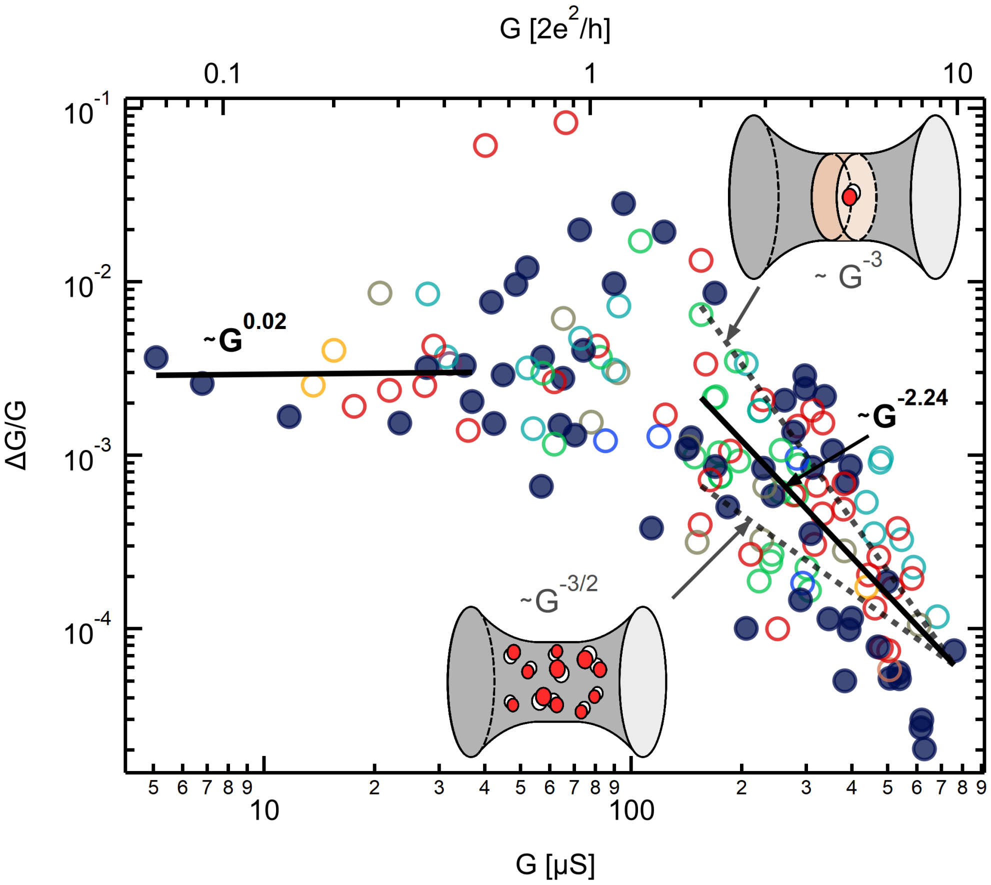

Figure S3 The conductance dependencies of the steady-state relative noise. Relative noise datasets are distinguished by different coloring corresponding to 9 independent $Ta/Ta_2O_5/Pt$ memristive devices. To highlight the trend on a single device, we have plotted the data for one device with filled blue circles, as opposed to the other devices where we have used open circles of different colors. Two distinct transport regions can be identified based on the fitted $\Delta G/G$ vs. G dependencies, shown with solid black lines. (i) A rather conductance-independent relative noise characteristic for the low-conductance tunneling barrier-like junction, and (ii) steep conductance dependence of the relative noise at $G > 2G_0$ conductances non-broken filamentary regime of a diffusive point-contact. The considered conductance-dependence limits are illustrated with gray dashed lines, while the insets demonstrate the respective illustration of point-contact geometries with fluctuators distributed in the entire volume and a single fluctuator in the narrowing.

There is another relevant situation, where also a diffusive point-contact is considered, but only a *single* fluctuator is placed around the device bottleneck (see upper illustration). In this case, the R_{PC} resistance of a point-contact is the sum of the resistances of slices, from which the light brown slice at the device bottleneck includes the single fluctuator, i.e., the ΔR_{PC} resistance fluctuation of the entire point-contact is the same as the $\Delta R_{\mathrm{slice}}$ resistance fluctuation of the slice containing the fluctuator. On the other hand, the slice can be considered as the parallel conductances of elementary volumes, from which only the elementary volume including the fluctuator fluctuates, i.e., the $\Delta G_{\mathrm{slice}}$

conductance fluctuation of the slice is the same as the $\Delta G_{\mathrm{fluctuator}}$ conductance fluctuation of the elementary volume including the fluctuator. Using the $\Delta R^2_{\mathrm{slice}} = \Delta G^2_{\mathrm{slice}}/G^4_{\mathrm{slice}}$ conversion between conductance and resistance fluctuations we can conclude in the $\Delta R^2_{\mathrm{PC}}/R^2_{\mathrm{PC}} = \Delta G^2_{\mathrm{PC}}/G^2_{\mathrm{PC}} = \Delta I^2_{\mathrm{PC}}/I^2_{\mathrm{PC}} = \Delta G^2_{\mathrm{fluctuator}} \cdot G^2_{\mathrm{PC}}/G^4_{\mathrm{slice}}$ relation. Note, that $\Delta G_{\mathrm{fluctuator}}$ does not depend on the junction diameter, whereas $G^2_{\mathrm{PC}} \sim d^2$ and $G^2_{\mathrm{slice}} \sim d^4$ relations hold, from which $\Delta G_{\mathrm{PC}}/G_{\mathrm{PC}} \sim d^{-3} \sim G^{-3}_{\mathrm{PC}}$ follows. See the cubed dependence indicated by the gray dashed guide to the eye line.

The steep conductance dependence of the relative noise in the studied Ta_2O_5 crosspoint devices at $G > 2G_0$ conductances can be fitted by a $\Delta G/G \sim G^{-2.24}$ relation (see black solid line in Fig. S3). This indicates, that the noise characteristics of the studied Ta_2O_5 crosspoint devices are not sufficiently described by either of the two limiting fluctuator distributions. This conclusion is surprising given that conductance dependencies of the relative steady-state noise of Ta_2O_5 STM point-contact devices followed the $\Delta G_{\mathrm{PC}}/G_{\mathrm{PC}} \sim G^{-3/2}_{\mathrm{PC}}$ conductance dependence of fluctuators distributed in the entire volume[3]. The above considerations imply that the increased contribution of a single fluctuator in the crosspoint devices could account for the distinct conductance dependencies of the relative steady-state noise when compared to the STM point-contact devices. Beyond the obvious differences in the STM point-contact and crosspoint geometry, the growth of the Ta_2O_5 layer in the former was performed by anodic oxidation in contrast to the reactive high power magnetron impulse sputtering in the case of the crosspoint samples. How the sample geometry and the oxide layer preparation impact the distribution of the fluctuator, is still an open question.

4 Cycle-to-cycle and device-to-device variation of relative noise preceding and during reset transitions

Here, we analyze the full-cycle noise variation, the cycle-to-cycle noise variation and the device-to-device noise variation in more detail. First, we replot the data from Figs. 2c1,c2,c3,c4 of the main text with the vertical axis highlighting the relative current variation expressed as a percentage. This figure clearly demonstrates (i) the highly enhanced relative noise levels at higher voltages ($V = -650\,\mathrm{mV}$ and $V = 650\,\mathrm{mV}$, see Figs. S4b,c, respectively), and (ii) the fundamentally different relative noise level at the same low readout voltage ($V = \pm 150\,\mathrm{mV}$) prior and after the switching cycle (see Figs. S4a,d, respectively).

Fig. 3b in the main text analyzed the huge cycle-to-cycle variation of relative noise of highly reproducible $I(V)$ curves. Here, in Fig. S5a,b, we elaborate on the conductance and relative noise evolution preceding and during the reset transition (negative voltage branch of the HCS) of the

same 10 subsequent cycles.

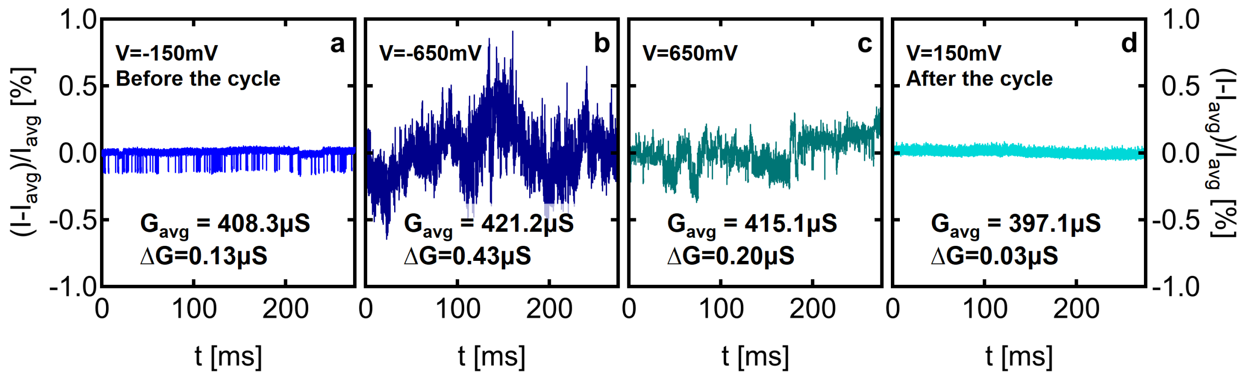

Figure S4 Relative current variation expressed as a percentage. (a-d) The data are replotted from Figs. 2c1,c2,c3,c4 of the main text (see panel a,b,c,d respectively) highlighting the relative current variation on the vertical axis. The voltage values together with the average conductance values and the standard deviation of the conductance are indicated on the panels.

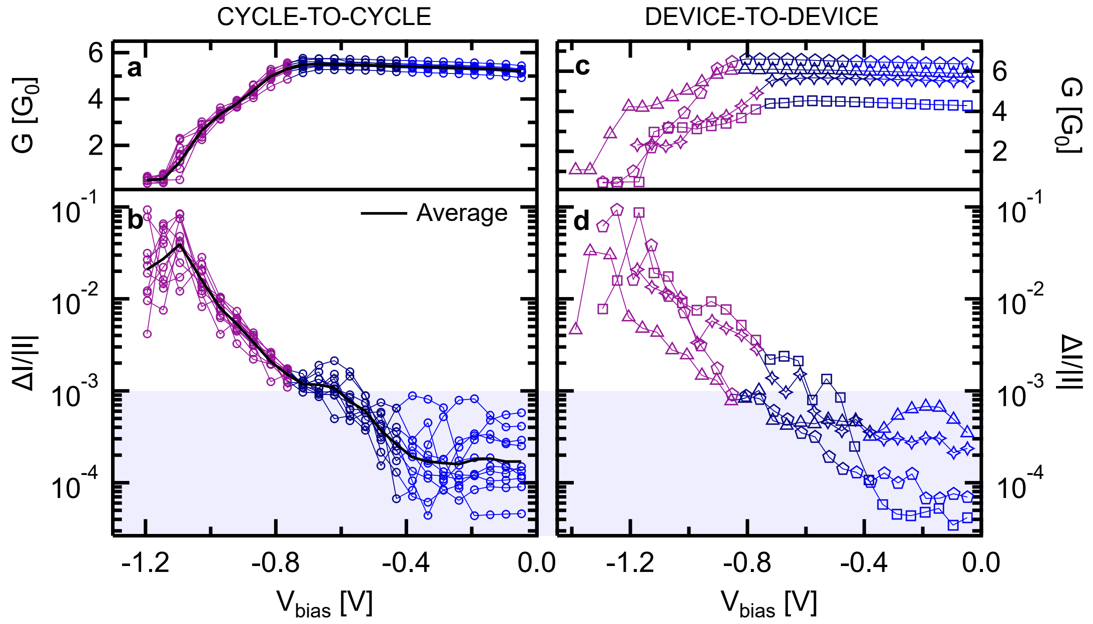

Figure S5 Cycle-to-cycle and device-to-device variation of the reset branch. (a-b) Zoom into the conductance (a) and relative noise (b) of the 10 subsequently measured cycles presented in Fig. 3a,b of the main text, with attention to the HCS at negative polarity where the reset transition happens. The color-coding of the individual curves is consistent with the steady state (blue), non-steady state (dark blue) and switching (purple) distinction, while the average curve is black. (c-d) Same evaluation presented on the individual reset branches measured on 4 different devices, all exhibiting HCSs in the range of $4.3 - 6.4\ G_0$. The measurements are colored based on the same distinct color-coding for the relevant regimes.

Each cycle is color-coded in the same fashion as explained in the main text, i.e., the blue, dark blue, and purple regions demonstrate the steady-state, non-steady state, and switching conduc-

tance regions. Additionally, the average curve is indicated with black color. Although there clearly is a cycle-to-cycle variation of the relative noise, the individual cycles follow a uniform trend consistent with the analysis of the average relative noise trends, exhibiting a similar decomposition to (i) a steady-state region (blue), where both the conductance and the relative noise are mostly constant; (ii) a non-steady-state region, where the conductance is constant but the noise increases immensely (dark blue) and (iii) the switching region (purple), where the conductance decreases while the noise further increases. As a comparison, Fig. S5c,d provide individual reset branches on 4 different devices (all exhibiting HCSs in the range of $4.3 - 6.4\ G_0$) while using the same color coding. These measurements imply that the observed trends are universal from device to device. Additionally, based on the relative noise map, it is not surprising that there is a rather high device-to-device variation of the steady-state relative noise which is obvious from the blue region of panel (d). However, this analysis clearly highlights that the steady-state noise of the highly reproducible resistive switching cycles also exhibits a remarkable cycle-to-cycle variation which is comparable to the device-to-device variation (blue region of panel (b)).

5 Reproducible noise characteristics of non-steady-state subthreshold cyclings

In the following, we present the relative noise with a non-monotonic voltage dependence during a non-steady-state cycling where the voltage-induced tuning of a dominant fluctuator is observed. In this example of the reproducible subthreshold cycling in Fig. S6, we demonstrate the evolution of the full frequency-dependent noise spectra as the voltage is varied to gain better insight into the particular voltage-dependence. The measurement is performed following the same subthreshold measurement protocol introduced in the main text. Panel (a) demonstrates the evolution of the noise as the voltage is ramped up (dark red) and down (red), and (b1)-(b9) depict corresponding noise spectra at selected V_{drive} amplitudes indicated on the right side and by the vertical dashed lines in panel (a). The relative noise exhibits an initial decrease before the characteristic increasing tendency of the non-steady state is observed. The underlying explanation can be understood by the analyzing the composition of the noise spectra.

Each noise spectrum is fitted by the sum of a 1/f-like spectrum (ensemble of more remote fluctuators) and a single Lorentzian (a single dominant fluctuator positioned close to the device bottleneck), see description in Section 2. in the Supporting Information. The cut-off frequency of the Lorentzian part is illustrated as a dashed vertical line. Initially, the steady-state noise has a

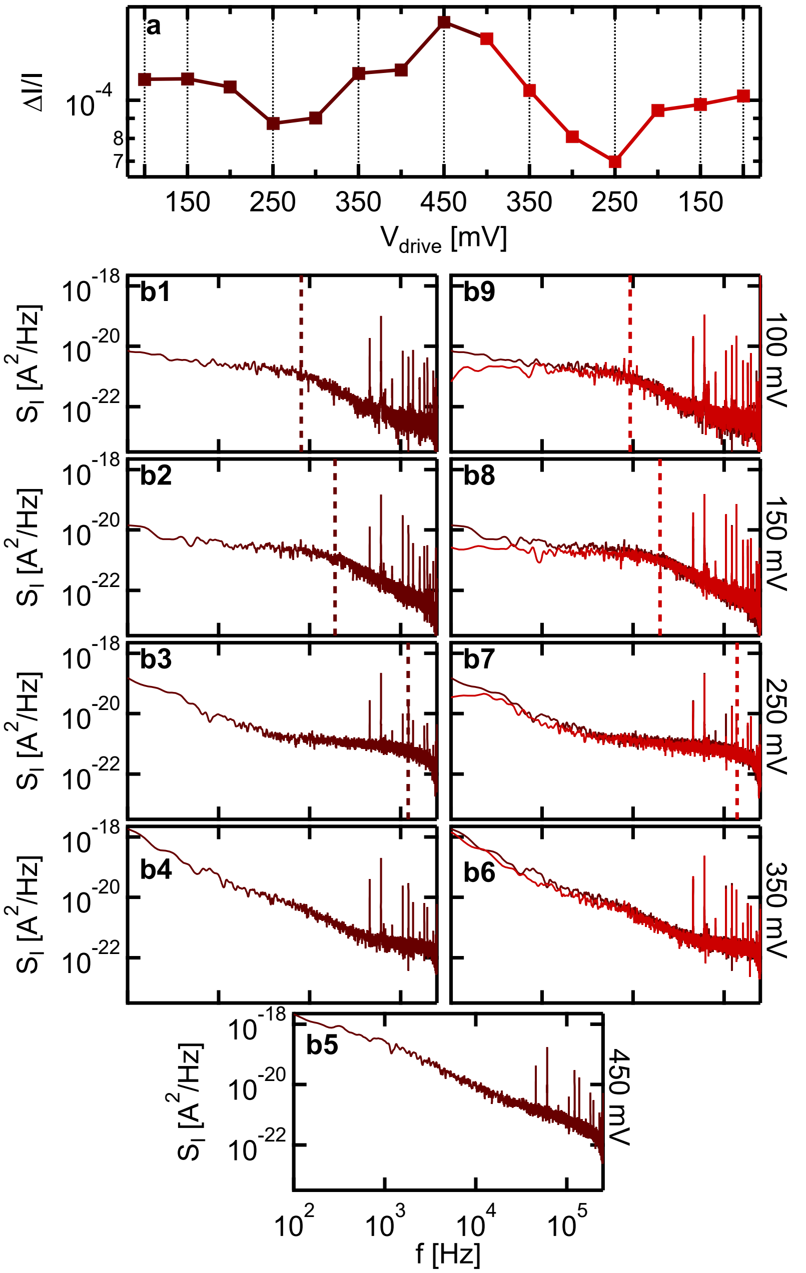

Figure S6 Extended analysis of a steady-state cycling with non-monotic voltage dependence of relative noise. (a) Voltage dependence of the total relative noise during the upward (dark tones) and the downward (light tones) ramp. (b1-b9) Noise spectra at selected drive voltages (label on the right) are plotted underneath the corresponding sweep with the same color coding for the downward/upward direction. Panels (b6)-(b9) include the upward sweep's spectra at the same drive voltages in the background as a comparison.

significant contribution from a single fluctuator which is evident from the dominantly Lorentzian-type spectrum in panel (b1). With increasing voltage, the Lorentzian amplitude is decreased and relaxation time is detuned, see the Lorentzian spectra shifting to a higher cut-off frequency and

eventually out of the integration window (panels (b1)-(b5)). The relative noise decreases correspondingly, and with the onset of the non-steady state, voltage-induced activation of a large number of fluctuators starts increase the contribution of a 1/f-type spectra to the noise. The non-monotonic voltage dependence is nicely reproduced in the downward ramp (see panels (b6)-(b9) consisting of the PSDs of both upward (dark red) and downward (red) ramp), i.e., the noise spectra reproduce at the same drive voltages and the corresponding decomposition to 1/f-like and Lorentzian spectra yield nearly identical proportions. This finding also confirms that the cycling in the non-steady state, applied voltages approach but do not reach the threshold voltage for switching.

References

[1] Balogh, Z.; Mezei, G.; Pósa, L.; Sánta, B.; Magyarkuti, A.; Halbritter, A. 1/f Noise Spectroscopy and Noise Tailoring of Nanoelectronic Devices. *Nano Futures* **2021**, *5*, 042002.

[2] Sánta, B.; Balogh, Z.; Gubicza, A.; Pósa, L.; Krisztián, D.; Mihály, G.; Csontos, M.; Halbritter, A. Universal 1/f Type Current Noise of Ag Filaments in Redox-Based Memristive Nanojunctions. *Nanoscale* **2019**, *11*, 4719–4725.

[3] Sánta, B.; Balogh, Z.; Pósa, L.; Krisztián, D.; Török, T. N.; Molnár, D.; Sinkó, C.; Hauert, R.; Csontos, M.; Halbritter, A. Noise Tailoring in Memristive Filaments. *ACS Applied Materials & Interfaces* **2021**, *13*, 7453–7460.

[4] Wu, Z.; Wu, S.; Oberholzer, S.; Steinacher, M.; Calame, M.; Schönenberger, C. Scaling of 1/f Noise in Tunable Break Junctions. *Physical Review B* **2008**, *78*, 235421.

[5] Maxwell, J. C. *A Treatise on Electricity and Magnetism*; Clarendon Press, Oxford, 1904.

[6] Halbritter, A.; Borda, L.; Zawadowski, A. Slow Two-Level Systems in Point Contacts. *Advances in Physics* **2004**, *53*, 939–1010.
